# Adherence, tolerability, and outcome after 36 months of isoniazid-preventive therapy in 2 rural clinics of Swaziland: A prospective observational feasibility study

**DOI:** 10.1097/MD.0000000000007740

**Published:** 2017-09-01

**Authors:** Yolanda Mueller, Qhubekani Mpala, Bernhard Kerschberger, Barbara Rusch, Gugu Mchunu, Sikhathele Mazibuko, Maryline Bonnet

**Affiliations:** a Epicentre, Paris, France; b Médecins Sans Frontières, Geneva, Switzerland; c National Tuberculosis Control Program; d Swaziland National Aids Program, Ministry of Health, Swaziland; e IRD UMI 233 TransVIHMI-UM—INSERM U1175, Montpellier, France.

**Keywords:** adherence, isoniazid preventive therapy, TB-HIV

## Abstract

Although efficacy of 36 months isoniazid preventive therapy (IPT) among HIV-positive individuals has been proven in trial settings, outcome, tolerance, and adherence have rarely been evaluated in real-life settings.

This is a prospective observational cohort study conducted in 2 primary care rural clinics in Swaziland.

After negative tuberculosis symptom screening, patients either with the positive tuberculin skin test (TST) or after tuberculosis treatment were initiated on IPT for 144 weeks. In addition to routine clinic visits, adherence was assessed every semester.

Of 288 eligible patients, 2 patients never started IPT (1 refusal, 1 contraindication), and 253 (87.8%), 234 (81.3%), and 228 (79.2%) were still on IPT after 48, 96, and 144 weeks, respectively (chi^2^*P* = .01). Of 41 patients who interrupted IPT before 144 weeks, 21 defaulted (of which 17 also defaulted HIV care); 16 stopped because of adverse drug reactions; 2 were discontinued by clinicians’ mistake and 1 because of TB symptoms. Five patients (1.7%) died of causes not related to IPT, 5 (1.7%) developed TB of which 2 were isoniazid-resistant, and 9 (3.1%) were transferred to another clinic. As an indicator of adherence, isoniazid could be detected in the urine during 86.3% (302/350) and 73.6% (248/337) of patient visits in the 2 clinics, respectively (chi^2^*P *<* *.001).

The routine implementation of IPT 36 months was feasible and good patient outcomes were achieved, with low TB incidence, good tolerance, and sustained adherence.

## 1 Introduction

In settings with high tuberculosis (TB) burden, isoniazid (INH) Preventive Therapy (IPT) is recommended for prevention of active tuberculosis in people living with HIV (PLHIV), particularly in individuals who are tuberculin skin test (TST)-positive.^[1–3]^ In addition to antiretroviral therapy (ART), IPT reduces the incidence of active TB^[4]^ and was initially recommended for 6 months.^[3]^ However, effectiveness of IPT has been shown to diminish 6 to 18 months after completion of such a regimen,^[5–7]^ suggesting that IPT should be provided for longer. Indeed, a trial conducted in Botswana showed that TB incidence among TST-positive PLHIV was reduced by a further 43% if IPT was given for 36 months as compared to 6 months,^[8]^ although the reduction was not significant in PLHIV with negative or unknown TST results.

Despite these results, uptake of 36 months’ IPT has been slow in many countries.^[9–11]^ Main concerns about long-term IPT were risk of side-effects, patient adherence, development of active TB while on IPT, and increased risk of INH-resistance. For example, in South Africa, up to 28% of cases suffered from a grade 3 or 4 transaminase elevation, after a median of one-and-a-half year on IPT and only 60% were retained on IPT at 3 years.^[12]^ Besides, although benefits of IPT are greatest in TST-positive patients, the introduction of widespread TST in resource-limited settings is limited by operational constraints such as cold chain requirements and the extra-visit required for TST reading. Although clinical trials have been essential to inform recommendations about 36 months IPT, there have been no observational studies documenting actual implementation. Confirming results from clinical trials in routine settings can help to convince decision-makers about the added value of 36 months IPT.

Even though TB incidence has decreased in recent years, Swaziland is still the country with the highest TB and HIV burden reported in the world.^[13]^ National TB control has focused on early detection and treatment of TB, in parallel with rapid scaling up and integration of ART. Although IPT was recommended for 6 months, its implementation remained challenging in 2011, and extending IPT duration to 36 months seemed unfeasible. There was also the concern that adherence and outcomes under routine conditions would be less favorable than those reported in clinical trials. Therefore, the objective of this study was to assess the feasibility of TST-based 36 months IPT strategy in HIV-infected patients in programmatic conditions in 2 rural clinics of the Shiselweni region in Swaziland, in order to inform policy-makers in the country and in similar settings. This paper focuses on adherence, tolerability, and outcomes after 48, 96, and 144 weeks of follow-up. The results of this study on initial patient screening, implementation of tuberculin-skin test, and initiation of IPT have been published previously.^[14]^

## 2 Methods

This prospective observational cohort included PLHIV eligible for IPT followed in 2 rural nurse-led primary care clinics in the Shiselweni region, Kingdom of Swaziland (OLOS clinic in the Hlatikulu zone and New Haven clinic in the Matsanjeni zone). The study was undertaken in compliance with the Declaration of Helsinki and was approved by the Social Welfare Scientific Ethical Committee of Swaziland and the Médecins Sans Frontières Ethical Review Board. Written informed consent was obtained from all study participants.

Details of study setting and screening process can be found elsewhere.^[14]^ Briefly, both clinics served an active ART cohort of about 700 patients. Nurses were in charge of providing HIV-TB care, which was fully integrated into general outpatient care. Expert clients (PLHIV trained to provide patient support education and counseling) assisted the nurses in managing patient files and clinic registers, as well as adherence support. Medical doctors visited the clinic once per week or every 2 weeks, according to needs, to attend to complicated cases and offer technical support.

The sample size was calculated to estimate the proportion of positive TST.^[14]^ The IPT study population, derived from the proportion of positive TST, consisted of all at-least-16-years-old PLHIV enrolled for HIV care at 1 of the 2 study clinics who were eligible for 36 months’ IPT based on either a positive TST (≥5 mm induration) or for secondary prophylaxis (after TB treatment^[2]^). Exclusion criteria were kept to a minimum to be as close as possible to the routine program conditions. According to national recommendations for IPT provision, patients did not receive IPT when they were on TB treatment, where TB was suspected, or when INH was clinically contraindicated (e.g. hepatitis and allergy). Informed consent was obtained before TST screening.^[14]^

Intensified TB case-finding was implemented at every contact between a PLHIV and the health facility, based on symptom screening (cough, night sweats, fever, weight loss, or chest pain) as per national recommendations.^[15,16]^ Diagnosis of TB was initially based on auramine-stained microscopical examination and XpertMTB/RIF (Cepheid, Sun Valley) testing performed at health-center level (secondary level), and later only on XpertMTB/RIF as per national recommendations.^[17]^ Samples from smear- or Xpert-positive patients were sent to reference laboratories at regional or national level for mycobacterial culture and drug susceptibility testing.

Patients were seen after 1 month after starting IPT. Subsequently, appointments followed the usual visit schedule for ART or cotrimoxazole refill, that is, every 1, 2, or 3 months depending on patient, clinic, and drug stocks. Laboratory monitoring followed the general recommendations for patients under HIV care in Swaziland, namely 6-monthly CD4, and quarterly liver function test. In 2012, viral-load testing was added to routine monitoring as per the national VL algorithm. Expert clients traced patients on HIV care by phone when clinical appointments were missed by 3 days. A renewed attempt was made by phone in cases where patients did not present at the clinic over a period of 90 days. IPT outcome definitions were adapted from the national standard definitions used to monitor 6 months’ IPT:^[16]^ “default” was defined as a patient who had taken INH for at least 1 month, then interrupted for 60 consecutive days or more; “failure” was defined as someone developing active TB disease while on IPT; “treatment discontinuation” was used for someone whose IPT had been discontinued by a health care worker due to adverse effects or any other reason; “transfer out” was used for someone who transferred to another ART site or region to continue treatment; “death” were those on IPT who were reported to have died of any cause during the course of treatment; “completed IPT” was defined as a full course of 36 times 4 weeks (total 144 weeks) of INH.

Pill count was systematically recorded at every patient contact. In addition, for all patients on IPT for 36 months, an in-depth evaluation was conducted twice yearly, consisting of a urine sample and a short questionnaire. Adherence was assessed through a combination of 4 parameters: pill count (i.e., the number of INH tablets remaining in the INH blister when coming for drug refill, including refill for antiretroviral or other concomitant drug), the self-reported number of days of no INH intake in the past 14 days, the Morisky scale, and isoniazid testing in the urine. For this, isoniazid strip tests (BD TAXO INH Test) were performed on urine samples following the manufacturer's instructions. This test is mostly reliable for the presence of isoniazid in the urine if the drug was taken in the last 24 hours.^[18]^ The Morisky scale^[19]^ combines 4 questions related to different aspects of drug intake by patients on chronic treatment (1. Do you ever forget to take your medicine? 2. Are you careless at times about taking your medicine? 3. When you feel better do you sometimes stop taking your medicine? 4. Sometimes if you feel worse when you take the medicine, do you stop taking it?). One point is attributed for every positive answer. Adherence is judged high, intermediate, or low for scores of 0, 1 to 2, and 3 to 4, respectively.^[19]^

Data were collected by the clinic staff on regular patient files during the routine follow-up visits, before extraction on study-specific case report forms by a study nurse. Occurrence of adverse drug reactions (ADR) was recorded in the patient file by the clinic nurse. Adverse drug reactions were defined as: “a response to a medicine which is noxious and unintended, and which occurs at doses normally used in man.” Additional information was requested in case of ADR, death, active TB, or treatment discontinuation. In addition, during adherence counseling, expert clients asked about side-effects experienced by the patients, without further precision on the definition of side-effect.

Data were entered on site weekly using Epi-Data 3.0 software (The EpiData Association, Odense Denmark), initially by a data entry clerk and later by the study nurse. Data were analyzed in Stata 12.1 software (College Station, TX). No imputation was done in the case of missing variables, apart from the date of death which was chosen randomly between date of last visit and date of information about deaths, in the absence of other information. Main outcome was described by the proportions that completed treatment, failed (developed TB), died, defaulted, discontinued, or transferred. The nonparametric test for trend across ordered groups was used to assess changes on adherence indicators over time. In the survival analysis of retention on IPT, death, TB, default and discontinuation were failures and transfers were censored. Predefined baseline characteristics that could represent risk factors for discontinuation (potential confounding factors), such as clinic, age, sex, ART status, initial baseline CD4 count at IPT initiation, and occurrence of adverse events, were tested in a non-parametric survival model, and rates of interruption per person-years were calculated. Variables associated with retention on IPT, defined as a log rank test <0.20, were further included in a multivariate Cox model in addition to age as a potential confounder.

## 3 Results

### 3.1 Patient characteristics

The study profile has been previously published.^[14]^ Patient characteristics are detailed in Table 1. Overall 288 patients initiated IPT for the following reasons: 217 tested positive at the first TST, 47 tested positive at the second repeated TST at 6 months after first TST, and 24 had completed TB treatment less than 6 months ago. Median age was 38 years (interquartile range [IQR] 31–47). The median time from HIV diagnosis to inclusion was 2.8 years (IQR: 1.4–4.8), with 252 patients (87.5%) already on ART. Median CD4 count at inclusion was 477 cell/microL (IQR 344 – 616). A total of 102 (35.9%) had been treated for tuberculosis previously, and 109 (38.5%) had previously received IPT for 6 months.

**Table 1:**
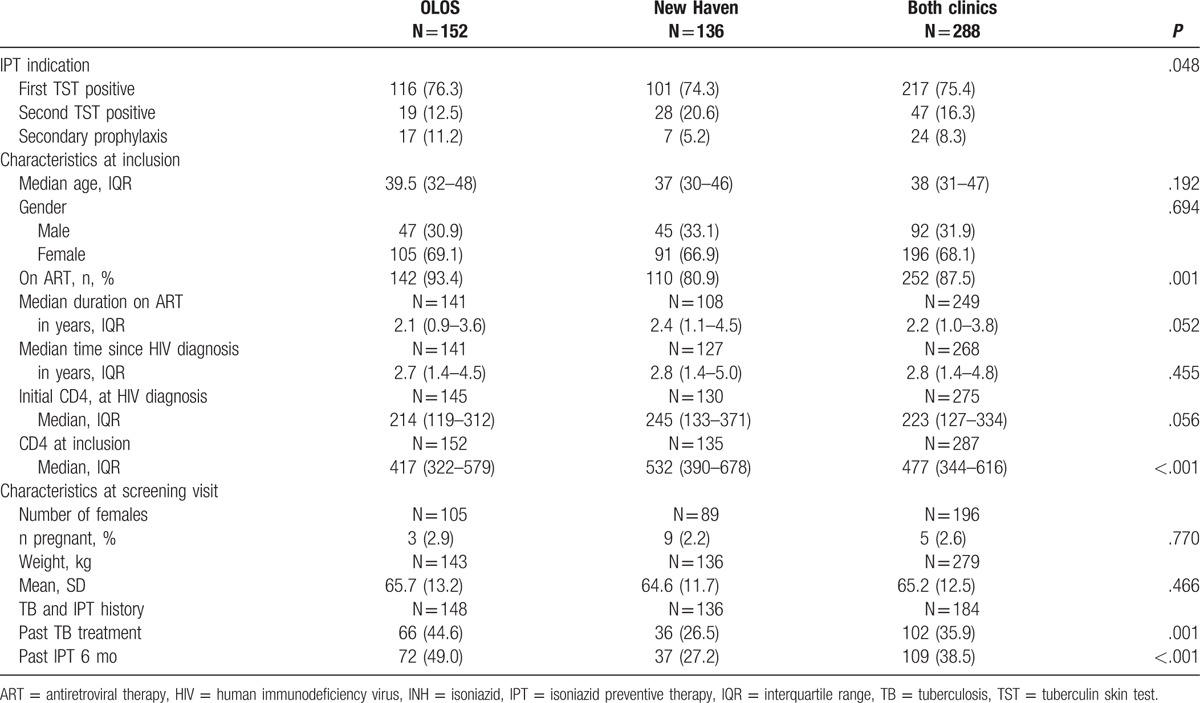
Characteristics of patients started on isoniazid preventive therapy, Swaziland, 2012–2015.

### 3.2 Outcomes

Out of a total of 288, 286 (99.3%) actually initiated IPT (1 refusal, 1 contraindication not previously recognized) and 253 (87.8%), 234 (81.3%), and 228 (79.2%) were still on IPT after 48, 96, and 144 weeks, respectively (chi^2^*P* = .01, Table 2). Of the 40 patients who did not complete IPT, 21 defaulted (of which 17 also defaulted HIV care), 16 discontinued IPT because of adverse drug reactions (detailed below), and 2 because of a mistake of the clinician, who stopped IPT after 6 months instead of 36 months. Nine patients (3.1%) were transferred to another clinic, and 5 (1.7%) started TB treatment (described in detail below). Finally, 5 patients (1.7%) died while on IPT, corresponding to a death rate was 0.7/100 person-years (5 deaths/702 person-years of follow-up; 95%CI 0.3–1.7%). Of those 5 deaths, there was 1 road traffic accident, 2 deaths of unknown cause, and 2 possibly due to TB (1 patient with symptoms compatible with TB meningitis, and 1 with important weight loss); none was judged possibly or probably related to IPT by the physician in charge.

**Table 2:**
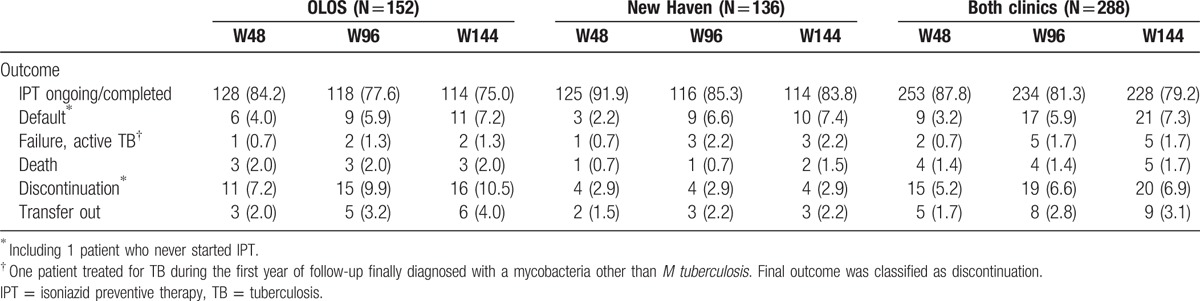
Cumulative outcomes after 48, 96, and 144 weeks on isoniazid preventive therapy, Swaziland, 2012–2015.

Five patients were diagnosed with TB over 702 person-years of follow-up, corresponding to an annual incidence rate of 0.7/100 person-years (95%CI 0.3–1.7/100PY). Among them, there were 2 new pulmonary drug-sensitive cases, 1 extrapulmonary case (TB of the spine diagnosed by radiography), and 2 patients infected with INH-resistant mycobacteria.

**Figure 1:**
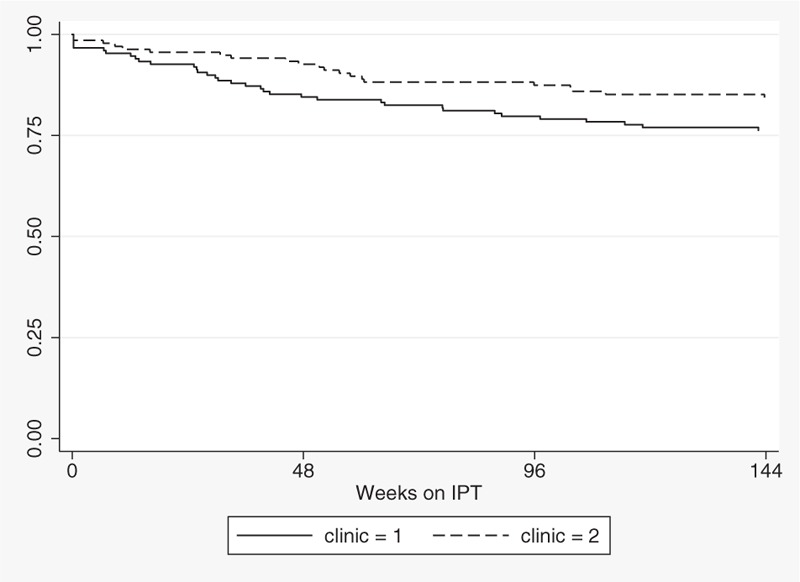
Kaplan–Meier curve of retention under IPT, by clinic.

Patients were followed up for a median of 149 weeks (IQR 146–163 weeks) after IPT start, for a total of 702 person-years (Fig. 1). In the adjusted non-parametric analysis (Table 3), gender and ART treatment were not associated with retention on IPT, while age, a high CD4 count at IPT initiation, absence of adverse event and clinic were associated with better retention. In a multivariate Cox model, occurrence of at least 1 adverse event was the strongest predictor of treatment interruption, whereas increasing age and higher initial CD4 level were associated with retention under IPT (*P* < .001 for goodness-of-fit test). The proportional hazards assumption was not violated (global test *P* = .325).

**Table 3:**
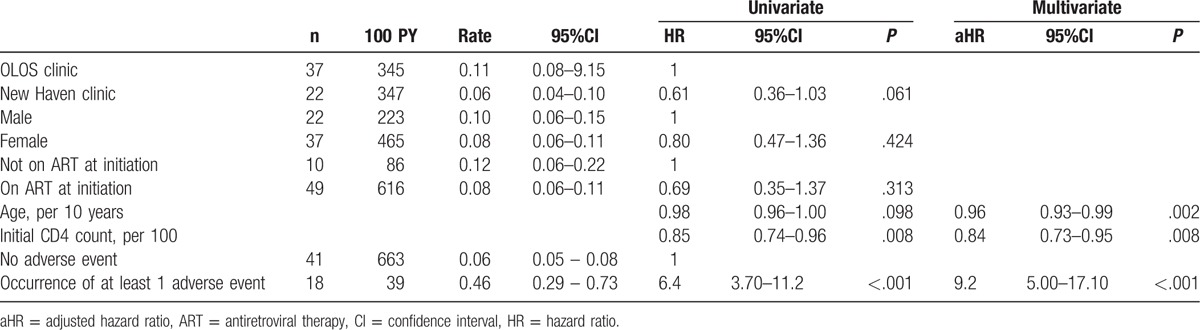
Univariate and multivariate Cox analyses of predictors of isoniazid preventive therapy interruption, Swaziland, 2012–2015.

### 3.3 Tolerance

Adverse drug reactions were mentioned during consultations by 5.6% (15/286), 2.0% (5/253), and 0.9% (1/234) patients during the first, second, and third year of follow-up, respectively. Three cases of hepatotoxicity were recorded (1.0%), of which 2 were severe (1 with ALT 5–10x, 1 admitted for jaundice) and 1 mild. All patients recovered. Other patients mentioned symptoms which may have been due to hepatotoxicity, such as abdominal pain (3 patients), or nausea and vomiting (2 patients, including 1 pregnant women). Unfortunately, IPT was often stopped without liver tests to confirm the diagnosis. The other reported adverse reactions were 2 cases of rash, 1 of itching, 2 cases of neuropathy, 3 cases of nonspecific weight loss, 1 psychotic decompensation, and 1 case of vertigo and nystagmus.

Treatment was discontinued for 16/22 patients reporting adverse-drug reactions. Of the 16 patients that discontinued IPT, 1 was found dead at home 2 months after IPT discontinuation, and 1 pregnancy resulted in stillbirth. The 2 patients with neuropathy recovered only partially. The remaining 13 patients were seen at a later appointment with no complaints raised.

Side-effects reported to the expert client during the adherence assessments decreased from 19.0% (16/79) and 50.0% (25/52) at 6 months in OLOS and New Haven, respectively, to less than 1% after 30 months. These side-effects were mostly itching, symptoms of neuropathy (tingling in hands or feet), nonspecific pain (joint pains), and other complaints (abdominal pain). None led to temporary treatment interruptions. Although the nurses reported few cases of neuropathy (1.4%), up to 4.9% (14/288) were reported during the interviews with the expert clients.

### 3.4 Adherence

Adherence was assessed at least once in 252 patients (87.5%), with a median of 3 assessments per patient (IQR 2–4). Urine was collected between 1 and 30 hours after reported INH intake (median 13 hours in OLOS and 5 hours in New Haven, ranksum test *P* < .001). Not all urine samples were actually tested, mainly because of the lack of available urine strips or of the staff either collecting the urine or performing the test. Of all assessments combined, the proportion of positive urine tests for isoniazid (INH) was 80.1% (95%CI 76.9–82.9%). Of the 17 patients who had the urine tested more than 24 hours after last INH intake, 14 (82.4%) were still positive. By contrast, out of the 141 negative urine tests, there were only 11 patients admitting no INH intake in the past 24 hours.

Although objective assessments such as the proportion of positive urine tests or pill count showed decreasing adherence over time (test for trend *P* = .04 and .12, respectively, Table 4), interview-based adherence indicators, such as the Morisky scale, or reported days of no intake seemed to improve over time. The main reason given for missed drug intake was not having the drug with them, or forgetting to take it. More rarely, patients cited lack of food, not feeling well, and running out of drugs before the appointment date.

**Table 4:**
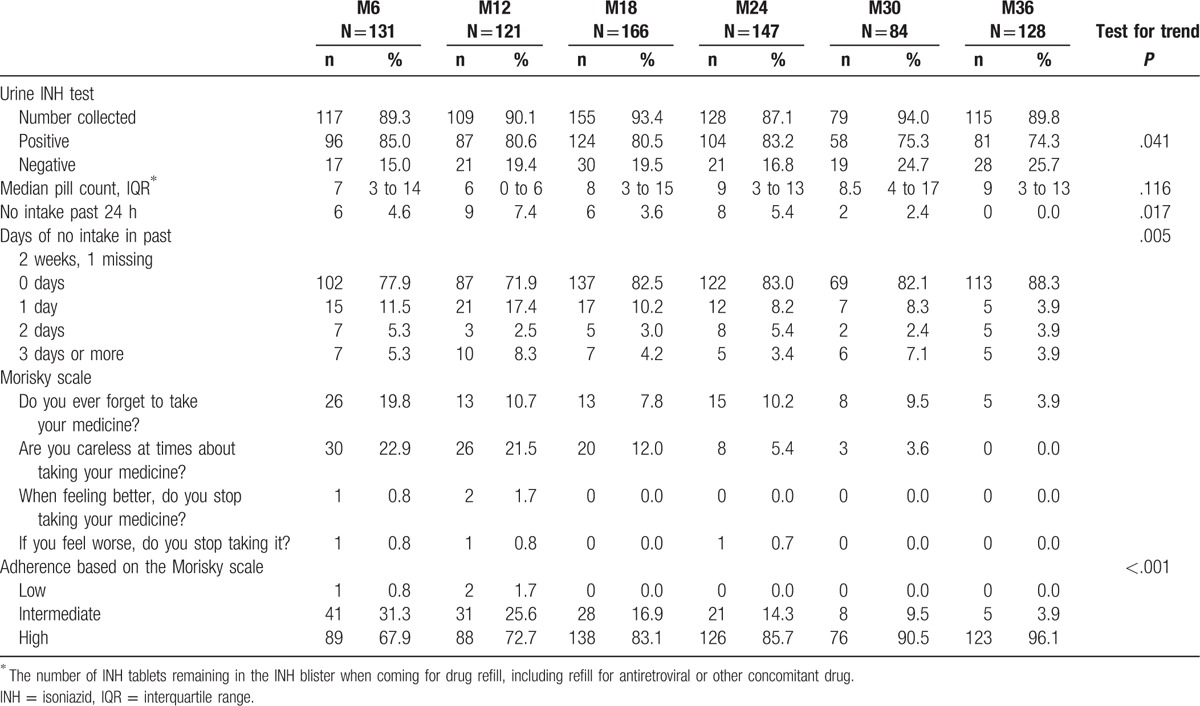
Adherence to isoniazid preventive therapy at M6, M12, M18, M24, M30, and M36, by pill count, intake in past 14 days, Morisky scale, and urine tests, Swaziland, 2012–2015.

## 4 Discussion

To our knowledge, this is the first study to document feasibility of 36 months’ IPT in the real life conditions of a high HIV/TB burden setting. In this setting, patient outcomes were similar or even more favorable than results obtained in African clinical trials that compared 36 months’ isoniazid with other IPT regimens.^[8,12]^ In particular, the incidence of TB in patients on IPT was comparable with other studies, even though Swaziland carries a high TB burden. However, most patients on IPT were already on ART for some time, had high CD4 counts, and therefore were at lower risk of TB than ART-naive patients. Two TB cases were due to isoniazid-resistant mycobacteria, which is not surprising in a setting were where 13.2% of new cases and 45.2% of previously treated cases harbor INH resistance.^[20]^ Interestingly, both age and a high initial CD4 count favored good retention on IPT, whereas the occurrence of adverse drug reactions led to treatment interruption.

Occurrence of adverse drug reactions, particularly hepatotoxicity, was comparable with findings from the Botswana trial.^[21]^ By contrast, there were more hepatotoxic events recorded in the clinical trial in South Africa^[12]^ than found in our study, maybe related to a higher prevalence of underlying hepatitis B and alcohol use. Also, liver tests were performed systematically in clinical trials, in contrast to our setting where, despite existing recommendations, systematic laboratory testing is rare. In our study, hepatotoxicity was more frequent during the first year on INH. In these cases, extending IPT from 6 to 36 months did not much increase the risk of hepatotoxicity. However, we cannot exclude substantial underreporting of adverse events in our study, considering that almost all adverse events reported led to treatment discontinuation. This may imply that some side-effects remained unreported.

Indeed, few cases of neuropathy were reported by the nurses given the number of reported instances of tingling, burning sensation, or pain in the hands and feet to the expert clients. Moreover, management of neuropathy was far from ideal, INH being often stopped instead of increasing vitamin B6. Training on these aspects should not be neglected during implementation of INH.

Adherence to INH was good, based on the results of the urine test, even if it slightly decreasing over time. However, some selection bias in adherence estimates is possible, as the patients who missed their appointment, and therefore urine testing, may also be the least adherent. Nonadherent patients were probably also more likely to have the drug discontinued because of vague side-effects, whereas the adherent patients were continuing on treatment. Still, our findings show that patients can be maintained on INH in routine conditions with good adherence. The main reason given by patients for missing a dose was not having the drug with them. This suggests that aspects of drug management while on travel should be reinforced during counseling. Also, differences in packaging between IPT and ART make dispensing a difficult task for health center staff. Coformulation with cotrimoxazole would certainly simplify IPT prescription.^[22]^

In the context of an observational cohort conducted in routine conditions, our study suffers from some limitations. First, we cannot exclude some selection in the study population because of nonexhaustive screening and/or TST refusal. Second, the study was conducted in only 2 clinics, which may not be fully representative of all the clinics in the country. Also, most patients were already ART-experienced, and results may not be generalized to contexts with many ART-naive patients. Third, we did not aim to compare outcomes of 36 months to 6 months or non-IPT provision, because the efficacy of IPT has been established by other studies.^[8,12]^ Also, the benefits and harms of short- and long-term IPT have been described previously and therefore were not evaluated.^[12,21,22]^ Finally, the quality of the information obtained for TB cases or adverse reactions was limited by the fact that many patients were not assessed, or only partly, by medical doctors. On the other hand, 1 strength was the limited number of missing data, considering the simple study set-up.

This feasibility study which was conducted in the public sector in a resource constrained setting shows that implementation of 36 months’ IPT under routine conditions leads to similar outcomes as have been demonstrated previously in clinical trials. Indeed, IPT given for 36 months was well tolerated, with a limited incidence of TB cases. IPT can easily be integrated into routine follow-up of HIV-positive patients. Furthermore, adherence can be maintained in the long run. These findings support the 2015 updated WHO recommendation of giving at least 36 months IPT to adults and adolescents living with HIV, who have an unknown or positive tuberculin skin test (TST) status, in resource-constrained settings with high TB incidence and transmission.

## Acknowledgments

The authors would like to thank the patients who participated in this study, the staff, and expert clients working in the clinics, and the entire MSF teams working in Swaziland. Special acknowledgment to Mathieu Bastard for statistical advice, to Clothilde Rambaud-Althaus for support in quality monitoring, and to Béatrice Vasquez and Calorine Mekiedje for help in data cleaning and project supervision.
